# A Qualitative Investigation of Characteristics Impacting Clinical Decision-Making in Integrated Behavioral Health Care

**DOI:** 10.1007/s11414-024-09891-6

**Published:** 2024-07-09

**Authors:** Ash M. Smith, Maria C. Prom, Lauren C. Ng

**Affiliations:** 1 Boston Medical Center, 720 Harrison Ave, Boston, MA 02118 USA; 2 Psychology Department, The Graduate Center, City University of New York, New York, NY USA; 3 Department of Psychiatry, Massachusetts General Hospital and Harvard Medical School, Boston, MA USA; 4 Psychiatry Department, Boston University School of Medicine, Boston, MA USA; 5 Department of Psychology, University of California Los Angeles, Psychology Building 1285, Box 951563, Los Angeles, CA 90095 USA

## Abstract

To support implementation of integrated behavioral health care (IBHC) models in local settings, providers may benefit from clinical decision-making support. The present analysis examines perspectives on patient characteristics appropriate or inappropriate for, and currently managed within, IBHC at a large medical center to inform recommendations for provider decision-making. Twenty-four participants (*n* = 13 primary care providers; *n* = 6 behavioral health providers; *n* = 5 administrators) in an IBHC setting were interviewed. Thematic analysis was conducted with acceptable interrater reliability (κ = 0.75). Responses indicated behavioral health symptom and patient characteristics that impact perceptions of appropriateness for management in IBHC, with high variability between providers. Many patients with characteristics identified as inappropriate for IBHC were nonetheless currently managed in IBHC. Interactions between patient ability to engage in care and provider ability to manage patient needs guided decisions to refer a patient to IBHC or specialty care. A heuristic representing this dimensional approach to clinical decision-making is presented to suggest provider decision-making guidance informed by both patient and provider ability.

## Introduction

Limited access to, and minimal engagement in, specialty behavioral health care upon referral from primary care has long been documented in the literature.^1–5^ Integrated behavioral health care (IBHC), a term that encompasses a large spectrum of mental health care models in which behavioral health providers serve patients from primary care settings,^6,7^ demonstrates improved referral, follow-up, and treatment initiation rates for behavioral health care in comparison to specialty behavioral health care,^2,8–10^ as well as high rates of engagement.^11^ A growing body of research demonstrates the superiority of IBHC models compared to “usual” primary care, including clinical outcomes, functioning, satisfaction, treatment adherence, and quality of life.^12–25^

Based on this evidence, IBHC has the potential to increase engagement in and effectiveness of behavioral health care across a variety of behavioral health conditions. As such, integrated models of care have been adopted widely in the USA.^26,27^ However, without the support and structure of grant funding and research protocols, IBHC models that demonstrate success in research settings may not be feasible and efficacious in real-world settings.^28–31^ While this “research-to-practice” gap^32–34^ is not unique to IBHC,^35^ bridging the gap by adapting and specifying the IBHC model to unique settings has the potential to improve care provision and support health outcomes.^28^

Provider clinical decision-making, primarily deciding whether a patient should be referred to integrated behavioral health or specialty care, is a particular area in which adaptation and specification may help bridge research-to-practice gaps. In research studies, providers are supported by highly structured, limited, and predetermined inclusion criteria for IBHC management, while providers in real-world settings are tasked with making clinical decisions without similar guidance.^6,7^ Primary care providers (PCPs) in real-world clinics may have insufficient training or experience assessing behavioral health conditions^36^ to evaluate behavioral health needs^37^ and severity in the context of IBHC programs^38^ without additional support.

In addition, patient participants in many IBHC research studies are typically higher functioning, have less severe and complex illness, and are less demographically diverse than populations served in real-world settings.^39^ Many studies limit inclusion to a single behavioral health condition, typically depression or anxiety disorders,^40^ and tend to exclude patients experiencing acute suicidality or those with comorbid psychotic, bipolar, trauma-related, or substance use disorders.^6,41–43^ Without research protocols that automatically refer patients with diverse conditions, complex illness, or emergent needs to specialty care, providers may lack clear guidelines for deciding whether a patient is appropriate for IBHC or better served in specialty behavioral health care.

In addition, some IBHC models rely on a stepped-care approach, in which patients are first managed within IBHC for low-intensity interventions and are then referred to specialty behavioral health care for higher-intensity intervention if indicated.^6,7^However, real-world IBHC clinics may not be equipped to manage more severe or complex behavioral health conditions at the first step of care. Rather, in many real-world care settings, some patients are managed within IBHC programs while others are directly referred to specialty care.^44^ This discrepancy between traditional stepped-care approaches to clinical decision-making (e.g., referring all patients to IBHC first) and real-world practice can contribute to the lack of guidance for providers.

Without clear guidance, providers must independently determine which patients are appropriate for management in IBHC and which patients should receive a referral to specialty behavioral health care. In local IBHC clinics, there are a myriad of factors that could influence provider decisions to manage a patient in IBHC or refer to specialty behavioral health care (e.g., burden of patients, availability of behavioral health interventions, access to behavioral health providers in IBHC versus specialty care, provider confidence in assessing behavioral health needs). Thus, the present analysis examines provider perspectives on patient presentations appropriate for management in IBHC or referral to specialty behavioral health care, within a real-world IBHC setting. Specifically, the authors leveraged a phenomenological approach^45^ to qualitative analysis in order to identify factors that influence provider decisions to refer a patient to IBHC or directly to specialty behavioral health care. The authors present their process and resulting decision-making model to provide an example to support clinical decision-making in local primary care settings moving toward the integration of behavioral health care.

## Methods

### Study Setting

Researchers interviewed providers and administrators in two adult primary care clinics housed within Boston Medical Center, a large, urban academic medical center which represents the largest source of safety-net care in the local area and surrounding communities.^46^ Data previously collected by Boston Medical Center characterized the patient population. Seventy-two percent of patients were considered underserved, including low-income and elderly populations.^47^ Seventy percent were people of color, and 33% were Black.^48^ Thirty-two percent of patients had a primary language that was not English,^47^ and about a quarter of families served experienced housing insecurity.^48^ Patients often presented to care with complicated life experiences, unmet psychosocial needs, and comorbid behavioral and physical health conditions.^48^

Patients served at Boston Medical Center were more demographically diverse,^39^ and experienced higher comorbidity^40^ and severity^39^ than those typically represented in research studies, which often have stringent inclusion and exclusion criteria. Given stark differences between the patient population served in IBHC at Boston Medical Center and those described in many IBHC research studies, this qualitative investigation may elucidate characteristics that inform provider decisions to refer patients to IBHC or to specialty behavioral health care while adopting IBHC models to serve real-world patient populations.

### Participants and Procedures

Researchers analyzed semi-structured interviews conducted with 24 participants (*n* = 13 primary care providers; *n* = 6 behavioral health providers; *n* = 5 administrators) working in IBHC at Boston Medical Center, a large urban academic medical center, over a 6-month interval in 2017.^49^ The process of adopting IBHC at Boston Medical Center began in 2013–2014. As discussed by Prom et al.,^49^ a lack of consistent practice in IBHC implementation limited the integration approach of the primary care clinics at Boston Medical Center. Notably, the local needs and available resources resulted in adaptation and integration of aspects from multiple different standardized IBHC models. As such, the result was a hybrid of multiple different models, including collaborative care models (CCMs) that involve collaboration between behavioral health teams and primary care physicians to provide stepped care management and coordination with specialists and community resources,^50^ co-located collaborative care models that integrate behavioral health teams within primary care clinics,^51^ primary care behavioral health (PCBH) models that integrate behavioral health clinicians as consultants into the primary care team,^52^ and screening, brief intervention and referral to treatment (SBIRT) models that involve behavioral health assessment and brief intervention in primary care followed by specialty care referral.^53^ Implementation of a hybrid IBHC model may provide an example of how IBHC implementation in real-world settings may differ from implementation in research settings, as the use of specific IBHC protocols may not be conducive to implementation in real-world settings with fewer resources.^49^

Providers and administrators who participated in the present research worked or provided consultation within IBHC programs in either General Internal Medicine (GIM) or Family Medicine (FM) primary care clinics. Providers and administrators included primary care physicians (PCPs), behavioral health social workers (BHSWs), psychiatrists, and nurse practitioners. Administrators had dual roles as direct clinical care providers, in addition to managing clinic setting and directing care implementation. PCPs and administrators screened patients for behavioral health concerns in primary care and referred patients to IBHC through the electronic system or through a “warm hand-off” to a behavioral health social worker (BHSW) or directly to specialty behavioral health care through the electronic system. For patients referred to IBHC, BHSWs conducted one 45-min assessment and provided three to five 30-min follow-up sessions or referred to specialty behavioral health care. Psychiatrists and nurse practitioners with primary roles within the specialty behavioral health (i.e., psychiatry) clinic at Boston Medical Center acted as consultants for PCPs or BHSWs within GIM and FM primary care clinics.^49^ Provider training in IBHC varied and depended on which supervisor completed provider on-boarding, the provider’s role, and the specific clinic.^49^ High provider turn-over contributed to heterogeneity in training.^49^ Additional details describing the implementation of IBHC within primary care clinics at Boston Medical Center are published elsewhere.^49^

Table 1 presents participants’ roles, settings, and genders. Due to the small number of BHSW and psychiatrist participants, the authors describe both BHSWs and psychiatrists as behavioral health providers to protect their confidentiality. Table 1 specifies the setting of participants’ primary role, such that behavioral health providers primarily based in the specialty behavioral health clinic were categorized within the specialty behavioral health setting, despite providing consultation within GIM and FM clinics. Participants were recruited by purposive sampling through email requests to all PCPs, behavioral health providers, and administrators within the FM and GIM IBHC programs. Sample size was determined in two ways, depending on role. Behavioral health provider and administrator sample size was determined by availability and agreement to participate. Informed consent was obtained from all providers and administrators who participated in the present research. A total of 41 behavioral health providers were contacted and 6 (14.6%) participated. All five (100%) administrators involved in IBHC participated. PCP sample size was determined by theoretical saturation. Ninety-two PCPs were contacted, and theoretical saturation was reached at 13 (14.1%) participants (12.9% of GIM PCPs, 18.2% of FM PCPs).

**Table 1:** Participant role, clinic setting, and gender

Role	*N* (%)
Primary care provider	13 (54.2)
Behavioral health provider	6 (25)
Administrator	5 (20.8)
Clinic setting	
General Internal Medicine	15 (62.5)
Family Medicine	6 (25.0)
Specialty Behavioral Health (Psychiatry)	3 (12.5)
Gender	
Female	17 (70.8)
Male	7 (29.2)

### Researcher Characteristics and Reflexivity

The researchers involved in the present analysis included one doctoral student in clinical health psychology, one psychiatrist with expertise in local and global behavioral health disparities, and one clinical psychologist with expertise in translational research for evidence-based intervention dissemination. At the time of the study, researchers held the following titles at Boston Medical Center: research coordinator, research fellow, and assistant professor. Two of the researchers are White Americans and one is Chinese American and African American. Two of the researchers are cisgender women and one is a nonbinary trans man. Researchers are invested in health equity, social justice, and anti-racism, and apply these values to their research broadly. Researchers used O’Brien et al.’s Standards for Reporting Qualitative Research (SRQR)^54^ to guide the current report.

### Interviews

Trained researchers, including a clinical psychologist, psychiatry resident, and two master’s in public health students, conducted interviews in-person or over the phone using a semi-structured interview guide. Interviews were audio-recorded and lasted 30 to 45 min. Researchers analyzed responses to questions about the characteristics, problems, or diagnoses that made a patient appropriate or inappropriate for IBHC, including: “Who are the patients who receive integrated care?”, “What characteristics of patients make them appropriate for integrated care? What types of problems or diagnoses are appropriate for integrated care?”, and “What patient characteristics, problems, or diagnoses make them inappropriate for integrated care?” The full interview guide was published elsewhere.^49^

Interviewers asked participants to describe the process of how a patient is referred to, managed by, and discharged from IBHC in their setting. Additional interview questions focused on current practices, purpose, definition, impact, benefits, and challenges of IBHC as implemented in participants’ clinics.^49^ Administrators with dual roles responded to interview prompts from their perspective as administrators or clinical providers. The Boston Medical Center and Boston University Medical Campus Institutional Review Board approved all study procedures, and all participants gave informed consent. Researchers secured data on audio-recording devices stored in a locked office, and password-protected servers.

### Thematic Analysis

Interviews were transcribed verbatim and coded using NVivo12.^55^ The coding team included two bachelor-level research assistants, two masters-level students, one medical student, one psychiatry resident, and one clinical psychologist. The coding team completed thematic analysis following Braun and Clarke’s^56^ six-phase method. The initial codebook was developed through consensus following preliminary independent coding, with its organization primarily based on interview questions. During the coding process, the coding team met regularly to revise and adapt the codebook until no new themes emerged and theoretical saturation was reached. After the coding was completed, codes were categorized into subthemes, and subthemes were organized into minor and major overarching themes. Themes were refined and revised, and data was recoded as necessary, ultimately resulting in primary, secondary, and tertiary themes. Six of 24 interviews (25%) were double coded. Researchers examined responses within the primary theme of Patient Characteristics, including subthemes (i.e., appropriate for IBHC, inappropriate IBHC, and currently managed in IBHC) to analyze factors influencing provider decision-making more inductively. The kappa coefficient and percent agreement for the Patient Characteristics primary theme were calculated using the NVivo12^55^ coding comparison and demonstrated acceptable inter-coder reliability (κ = 0.75; percent agreement = 96.47%).

## Results

There were three main subthemes identified within the Patient Characteristics theme data: appropriate for IBHC, inappropriate for IBHC, and currently managed in IBHC. Within each of these subthemes, participants described two main types of characteristics (Table 2): "behavioral health condition or symptom characteristics" and "patient characteristics." "Behavioral health condition or symptom characteristics" included behavioral health condition or symptom type (e.g., psychotic disorders, depression, anxiety), case complexity, duration of care needs, chronicity or acuity, severity, provider comfort-level with managing the condition or symptom, and stability. "Patient characteristics" included likelihood of engagement, sociodemographic characteristics, and patient preference. Table 2 presents each characteristic with an exemplar participant quote and whether the primary analysis coded it as appropriate, inappropriate, or currently managed in IBHC (see Table 2). Table 3 provides a summary of participant perspectives on how each characteristic impacts referral appropriateness and decision-making in primary care clinics.

**Table 2:** Thematic analysis characterization of patient characteristics: Exemplar quotes of characteristics influencing provider decision-making

Secondary themes	Exemplar quote	Primary subthemes		
		A^a^	I^b^	C^c^
*Behavioral health condition or symptom characteristics*				
Behavioral health condition or symptom type	“… The only people … [inappropriate for IBHC are] people who have extreme personality disorders … Even those we’ve tried … to get in, they may just kind of abuse the system and stuff like that. Or, take too much energy out of the system.” [PCP, GIM]		x	
	“I think the easiest answer [to what characteristics of patients make them appropriate for IBHC?] are the situational depressions, right? Somebody passed away, so it’s grief management and education, trying to stave off those depressive symptoms, keeping them engaged with friends and family, keeping them active, either outside, or outside the home at least, … giving them uplifting and pleasant activities to engage in. Also, situational anxiety, something like school. Lots of students come in and say, ‘I’m super worried about this test. I’ve been anxious for months. What do I do?’ So, we do the relaxation, the grounding, the meditation stuff with them. Even some, like, acute PTSD. So, like someone was in a car accident. We can work with the symptoms that they’re experiencing from that car accident.” [BH provider, GIM]	x		x
Case complexity	“So, treating basic anxiety, depression, insomnia, sort of acute stress things with short-term therapy, those are all fine. But once you get people with other comorbid illnesses or illnesses with some psychotic features or bipolar and so forth, then I don’t necessarily think I should [treat in IBHC] unless there is just no availability of psychiatry, but we have a patient population with high burden with fairly serious psychiatric disease. It’s not uncommon for people to have multiple comorbid psychiatric diagnoses.” [PCP, GIM]	x	x	x
	“It’s meant for patients with short- with … more straightforward issues. It’s meant to help manage them primarily in primary care. And it’s meant for patients with more significant issues to get them more accessible mental health treatment and support the PCP while they’re here.” [Administrator]	x		x
Duration of care needs	“I think the people that fund that category [appropriate for IBHC] are people that have some real acute stressor but don’t have [a] long history of depression, anxiety, substance abuse. People that are going about their life and they have some major catastrophic event, they are in crisis, they see somebody, probably would have recovered eventually anyway, but probably facilitated by seeing a therapist, and then get back to their baseline, which is relatively normal. The patients who have bipolar disorder or substance abuse along with it you know sort of other comorbid illness and things, I think they probably need longitudinal care in general and [we] just don’t have great longitudinal care here [in IBHC].” [PCP, GIM]	x	x	x
	“Any patient with primary care is potentially our patient. How long we engage with them or to what capacity we engage with them is where you might see some differences. Meaning we might not engage patients in short-term treatment if they have chronic mental health issues because what they really need is long-term care. So those are the patients we would briefly engage with, then move along to higher levels of care. Those patients include patients with schizophrenia or psychotic disorder, bipolar disorder, severe depression resulting in psychiatric hospitalization or suicide attempts. But then there’s a small set of even those chronically mentally ill patients for various barriers, we end up retaining longer—they become kind of our long-term panel.” [Administrator]			x
Acuity versus chronicity	“I think when someone is acutely psychotic or suicidal or homicidal, like all the extremes, I think they need something way more intensive than integrated care. So, either I have them go to an acute care setting or I get them urgently into psychiatry or we use the [emergency services] team, which is for taking care of people who are in real crisis, mental health crisis.” [Administrator]	x	x	
	“So, anything in the moment. Also, again, the managed chronic illness. So, we have people with bipolar disorder, people with schizophrenia, even people with personality disorders, who are aware of their symptoms, know their baseline, and just want to make sure that they have some new strategies, or some new interventions to take home and put in their bag. So, I think folks who, again, are acute in the moment, and folks who are well managed are great for integrated care.” [BH provider, GIM]	x		x
Severity	“Things that I think are inappropriate—for me, like psychosis, like schizophrenia, depending on the severity because I have a couple patients who are very functional and they’re schizophrenic.” [PCP, FM]	x		x
	“People with some severe mental illness … I think they are not necessary going to benefit from [IBHC] as much, though I think theoretically you can have someone working with a lot of case management and a lot of connection to medication and stuff like that and they might benefit from it.” [PCP, FM]	x	x	
Provider comfort-level with management of condition	“I think that care was particularly challenging and had aspects that are actually requiring advanced skills and training in a specific area, interacting with a nonverbal person and their guardian. …. so that’s not a typical case. I think maybe because … I feel relatively comfortable [with] a pretty broad array of psych care, that maybe I’m also not a typical case because I’m less apt to refer.” [PCP, GIM]		x	
	“I think generally speaking, our social workers service feel that something isn't in their scope of practice they are pretty good in referring people to those other services. I saw a guy who had concerns about his issues, violence, perpetrating partner violence, anger issues, and that was something I don’t think our social workers were comfortable with addressing so she made the appropriate refer to an anger management group at another location. So usually I think that our social workers are clear about what they feel comfortable on handling and if they are not clear or comfortable, they are able to refer to an appropriate resource.” [PCP, FM]		x	
Stability	“Psychotic or probably poorly controlled bipolar [are inappropriate conditions for management in IBHC]. Someone who needs medication management. But if, for instance, they’re seeing [psychiatric nurse practitioner] and she’s doing the medication and they’re managing and they’re under control, then that’s probably okay. […] Schizoaffective or schizophrenia […] or people who go in and out probably need to be under more intensive care.” [PCP, GIM]		x	x
	“People who I know are going through an acutely traumatic event but are generally very stable whether it’s grief or loss or you know something happening in their lives where I know that short term therapy will be beneficial, and that they’re sort of middle of the road in terms of their diagnoses and it’s certainly we can deal with moderate depression but nothing acute, right.” [Administrator]	x		
*Patient characteristics*				
Patient engagement	“It’s harder to know what to do with patients who’ve had a diagnosis of bipolar disease their whole life and nothing’s really changed acutely, but they’re just not well managed. I think those patients need to be in chronic behavioral health care, but I get them into that through the integrated behavioral health program because I think they’re more likely to engage that way. Rather than me trying to send them into this black box, in [specialty BH care]. Those patients probably benefit because I think they get engaged faster, but they are not going to actively managed in that setting.” [PCP, GIM]	x		
	“In terms of the characteristics of patients, any patient in whom I feel their psychiatric issue is kind of risen to the front, that they are ready to engage in care, that it is impacting, as it often does, their medical issues or their well-being, anyone that I assess in any way shape or form is ready to engage in care, I’ll refer them [to IBHC].” [PCP, GIM]	x		x
Sociodemographic characteristics	“I think everyone could benefit from this. The patients—I think the higher risk patients meaning patients who have unstable housing or unstable transportation—kind of the social stressors. It is easier for them to access services here.” [BH provider, FM]	x		
	“Refugees—we do see a lot of people that are new immigrants or refugees but the integrated behavioral health team—the traditional team—will actually send those patients to our refugee psychotherapist whose here on Thursday afternoons. Because they come with a lot of other complexities and issues regarding rape, torture, trauma, a lot of very important nuances of the countries they come from, what’s culturally adequate etc. They are more hardwired to be able to pick up on all those things, the refugee team, than actually our regular behavioral health. So they’re part of the family, but that part of the population we actually turf to the refugee team.” [Administrator]		x	
Patient preference	“I had another patient today who I saw who I’ve intermittently given her [benzodiazepines] for an anxiety condition. And I don’t think that it’s at all likely that she would be willing to, I mean she said, ‘I won’t go to Psychiatry for care.’ In theory she is someone who maybe could get support from the psychiatrists in GIM. This is maybe like on the phenotypes that integrated can help if people feel the stigma to go to the psychiatrist in a psychiatry clinic.” [PCP, GIM]	x		
	“I have a patient who is moderately depressed. I know I am not doing her right all the way, because I just know she needs talk therapy, but she refuses to see anybody else. She only wants to talk to me. So, I see her more frequently but it’s not enough. I have been struggling to figure out how to get her to trust somebody else, but it’s so entrenched in her- she has so much trauma, that she feels like she couldn’t possibly share this, any of this stuff with somebody else. […] You know, it’s not for everybody but it could benefit everybody.” [Administrator]	x		

*Notes. ADHD* attention deficit hyperactivity disorder, *BH* behavioral health, *FM* family medicine, *GIM* general internal medicine, *IBHC* integrated behavioral health care, *PCP* primary care provider

^a^Appropriate for IBHC

^b^Inappropriate for IBHC

^c^Currently managed in IBHC

**Table 3:** Thematic analysis characterization of patient characteristics: Summary of characteristics influencing provider decision-making

	*N*^a^	Appropriate for IBHC	Inappropriate for IBHC	Currently managed in IBHC
*Behavioral health condition or symptom characteristics*				
Behavioral health condition or symptom type	22	Acute stress, adjustment disorder, bereavement, eating disorders, OCD Mixed responses: depression, anxiety, PTSD substance use, ADHD, schizophrenia and other psychotic disorders, bipolar disorder, personality disorders	Mixed responses: ADHD, schizophrenia and other psychotic disorders, bipolar disorder, personality disorders Some providers say no conditions are inappropriate, but some are less indicated for IBHC	Depression, acute stress, anxiety, adjustment disorder, bereavement, substance use, schizophrenia and other psychotic disorders, bipolar disorder
Case complexity	20	Straightforward conditions or presentation, limited comorbidity (i.e., depression, stress, anxiety, insomnia, eating disorders, OCD) Some comorbidity (i.e., SUD, depending on provider comfort-level) Comorbid physical and behavioral health conditions	Comorbid behavioral health conditions (including SUD, personality disorder), conditions with psychotic features, complex PTSD, complicated patient presentation, diagnostic complexity, complex psychosocial needs	Mixed; more frequently managing straightforward cases, comorbid physical and behavioral health conditions, sometimes comorbid SUD depending on provider comfort-level
Duration of care needs	18	Short term (i.e., acute stress, situational anxiety, grief, adjustment disorder)	Long term (i.e., remittent depression, schizophrenia and other psychotic disorders, bipolar disorder)	Both; mainly short term, though also a “long-term panel”
Acuity versus chronicity	17	Acute, situational distress or symptoms (i.e., acute stress, anxiety, grief, adjustment disorder) Chronic, stable conditions (i.e., low severity SMI)	Chronic conditions that indicate needs for long-term management (i.e., SMI) Acute crises that IBHC is not equipped to manage (i.e., active suicidality/homicidality, psychosis)	Both; emphasis on acute behavioral health distress, but also addressing well-managed chronic behavioral health needs
Severity	14	Low severity, mild-to-moderate symptoms (i.e., depression, stress, anxiety, insomnia)	High severity (i.e., major depression, SMI, personality disorders)	Both; mild, moderate, and severe symptoms or conditions
Provider comfort-level with condition or symptom management	9	Comfort/training or experience in managing condition (i.e., depression, anxiety, SUD)	Limited comfort/training or experience in managing condition (i.e., ADHD, SMI, psychotic symptoms, SUD)	Comfort/training or experience in managing condition Mixed responses on comfort with managing various conditions (i.e., SUD)
Stability	6	Well-managed, stable conditions—even if chronic or indicative of long-term needs (i.e., well-managed SMI)	Patients who go in and out of care, need for medication management (i.e., SMI, severe personality disorders)	Stable conditions are managed in IBHC. Patients who have symptoms that are not well managed/stable may be triaged through IBHC, but are then referred to specialty care
*Patient characteristics*				
Patient engagement	13	Poor history of engagement in specialty care, higher likelihood of engagement in IBHC	High motivation to engage in specialty behavioral health care	Patients who are more likely to engage in IBHC than specialty care
Sociodemographic characteristics	6	Patient has sufficient resources, so they do not need additional resources or support Patient has limited resources, therefore needs the support of IBHC or is unlikely to engage in specialty care	Patient has limited resources and needs extra support Patient has sufficient resources to engage in specialty care	Mixed; patients with significant barriers to care and high burden of psychosocial needs, patients with sufficient resources who do not need additional resources or support
Patient preference	4	Patient preference for behavioral health care through primary care	Patient preference for referral to specialty care through outpatient psychiatry or community-based behavioral health care provider	Not enough information

*Notes. IBHC* integrated behavioral health care, *PTSD* posttraumatic stress disorder, *OCD* obsessive–compulsive disorder, *SUD* substance use disorder, *ADHD* attention deficit hyperactivity disorder, *SMI* serious mental illness

^a^Refers to how many participants (out of *N* = 24) mentioned the theme in their interview

### Appropriate for IBHC

Participants reported a variety of perspectives on which behavioral health condition or symptom characteristics and which patient characteristics were appropriate for IBHC (regardless of whether they were currently being managed in IBHC). Situations and conditions consistently reported as appropriate for IBHC management included grief and situational or acute symptoms of anxiety or depression or other acute distress. Obsessive compulsive, eating and feeding, adjustment, and sleep-related disorders were also mentioned as potentially appropriate for management in IBHC. Chronic or moderate/severe depression and anxiety, posttraumatic stress disorder (PTSD), substance use, and neurocognitive disorders received mixed responses (i.e., some participants described these as appropriate for management in IBHC, while others thought they were not appropriate for IBHC). In addition, some participants described psychotic and bipolar disorders as appropriate for IBHC, depending on the context, while others reported they would not be appropriate (Table 3).

The behavioral health condition or symptom characteristics and patient characteristics that influenced providers’ determination of appropriateness for IBHC were interrelated. For instance, some providers and administrators reported that a patient with a more chronic, severe, or complex psychotic disorder could be considered appropriate for IBHC if their condition is stable, if the patient was unlikely to engage in specialty care, or if the provider felt comfortable managing the particular condition.

### Inappropriate for IBHC

Participants reported a variety of perspectives on which condition or symptom characteristics and patient characteristics were considered inappropriate for management in IBHC (regardless of whether they were currently being managed in IBHC). Participants often considered psychotic and bipolar disorders (often described as “severe” or “serious mental illness”) inappropriate for IBHC, although some thought that stable or less severe psychotic or bipolar disorders could be managed in IBHC. Participants expressed that severe or chronic depression and anxiety were potentially inappropriate for IBHC. Participants typically considered suicidality as high risk. Some participants stated that this risk could be managed in IBHC; however, most reported that after the acute risk was addressed through IBHC, these patients should follow-up in specialty care. As noted earlier, some participants described substance use disorders, PTSD, and neurocognitive disorders as inappropriate for management in IBHC, while others thought these could be appropriate (Table 3).

In addition, characteristics that influenced providers’ determination of inappropriateness were interrelated. For instance, a patient with mild symptoms and a straightforward presentation may still be deemed inappropriate for IBHC if they have the resources, or a preference, to engage in specialty care. Similarly, participants described substance use as potentially inappropriate for IBHC if it was comorbid with another behavioral health condition, if the provider was not comfortable monitoring or prescribing medication management for substance use, or if the patient has limited resources. Neurodevelopmental disorders, such as autism spectrum disorder (ASD) or attention-deficit hyperactivity disorder (ADHD), also received mixed responses based on case complexity or provider comfort-level.

### Currently Managed in IBHC

Participants consistently reported the following conditions as being currently managed in IBHC: depression, acute stress, anxiety, adjustment disorder, bereavement, and substance use disorders. Some participants reported that psychotic and bipolar disorders were not currently managed in IBHC, although several providers mentioned a “long-term panel” of patients with chronic conditions or “serious mental illness” managed in IBHC. The characteristics that participants reported made a condition appropriate or inappropriate for IBHC were frequently inconsistent with those reported as currently being managed in IBHC, as demonstrated in Tables 2 and 3. Several participants reported that any patient with behavioral health needs could be referred to IBHC, even if only to facilitate referral to specialty care.

## Discussion

There is research support for IBHC across diverse conditions including anxiety and depression,^57–59^ PTSD,^60^ substance use,^61^ chronic pain and opioid use disorders,^62,63^ and psychotic and bipolar disorders.^64–67^ However, the current findings suggest the typical condition-based referral models within IBHC research studies may not represent the complexity of referral decision-making in primary care settings that differ from those frequently represented in IBHC research. The existing guidance that some IBHC models provide may be sufficient for settings that mirror those IBHC research studies or existing standardized IBHC models, like the primary care behavioral health (PCBH) model.^27,68–71^ However, in clinical care settings newly developing and implementing IBHC to serve diverse and complex patient populations, the findings indicate that PCPs and behavioral health clinicians would benefit from additional support in making clinical decisions about whether a patient is best served in IBHC or specialty care.

Providers in the GIM and FM clinics at Boston Medical Center had an overwhelmingly positive response to IBHC and believe that IBHC helps better serve patients.^40^ However, there were notable discrepancies between the patient characteristics described as currently managed versus those described as appropriate for management in IBHC. The present analysis also elucidated the heterogeneity between providers the factors that influenced whether a patient or behavioral health condition was appropriate for management in IBHC. This suggests that IBHC providers could benefit from adaptation and specification of the IBHC model to support clinical decision-making.

Participants identified multiple behavioral health conditions or symptoms and patient characteristics that influence whether a patient is adequately managed in IBHC versus specialty care. This included condition or symptom type, case complexity, duration of care needs, chronicity or acuity, stability, severity, provider comfort-level with managing the condition or symptoms, likelihood of patient engagement, sociodemographic characteristics, and patient preference. A number of these characteristics are consistent with existing literature, both in IBHC and more broadly. For instance, previous research on collaboration between primary and specialty behavioral health care suggests that PCP decisions on whether to manage depression themselves or refer to specialty care depends on severity, complexity, and their own comfort-level with management.^72^ IBHC research also indicates that the severity of depression symptoms impacts the setting in which a patient demonstrates long-term clinical improvement.^73^

In addition, some participants reported that IBHC would not be able to provide the necessary support to adequately serve patients struggling with substance use or limited resources. Individuals diagnosed with substance use disorders, unmarried people, and people of color demonstrate lower engagement in IBHC compared to those not diagnosed with substance use, married people, and white people.^74^ Bias, discrimination, and other structural barriers experienced by patients who use substances, are unmarried, and are not white may drive lower engagement across settings. Findings on IBHC engagement for individuals struggling with housing instability^75,76^ suggest the feasibility of IBHC for similarly minoritized populations, although more information on its effectiveness is needed.

Given IBHC’s ability to increase engagement in behavioral health services,^2,8–11^ patients managing substance use or limited resources (e.g., low-income) would likely benefit from referral to IBHC over specialty care. The contradictory intersection between patient needs and provider ability to manage patients with substance use or limited resources suggests a particular need for standardization and interventions to ensure that the patients who would benefit most from IBHC are not instead referred to specialty care. Because previous training experiences and implicit biases may inhibit provider ability to assess and treat patients in IBHC, standardization of clinical decision-making and training in behavioral health assessment and interventions may support equitable and effective IBHC implementation.

There were inconsistencies in the conditions participants deemed appropriate versus inappropriate for IBHC, and in the characteristics that influenced their referral decision-making. Given the overlap between patient characteristics reported as currently managed and inappropriate for management in IBHC, these inconsistencies likely exist in real-time referral decisions. Without clear guidance about referrals appropriate and inappropriate for IBHC, referral decisions may be based on clinic-specific characteristics rather than existing empirical evidence on the referral decision that will best meet a patient’s unique needs. Thus, heterogeneity in referral decision-making could explain discrepancies between positive IBHC research outcomes and inconsistent findings in real-world implementation.^8–31^ Standardization of referral decision-making may clarify which patients and what conditions are best managed in IBHC.

Furthermore, the current findings suggest that standardization of referral decision-making should be tailored to dimensional, rather than diagnostic, approaches to referral decision-making. It seems that the more confident providers are in their ability to manage patients within IBHC programs (based on skill, time, clinic resources), the more likely they are to manage them within IBHC. Provider confidence seems to relate more consistently to a provider’s perceived ability to manage the complexity of the case in front of them, rather than the diagnosis. Despite the high frequency of responses related to behavioral health condition or symptom type, high variability in whether providers deemed specific conditions or symptom types appropriate or inappropriate for management in IBHC suggests that condition-based referral decision-making in IBHC may be primarily driven by the interaction between condition complexity and provider confidence in their ability to manage the condition in IBHC, given the extent of patient and setting resources.

Participant responses often demonstrated how interactions between patient and provider characteristics impact referral decisions. For example, some respondents reported that patients with limited resources may be less likely to engage in specialty care and are thus indicated for IBHC, while those with sufficient resources are less indicated for IBHC because they are more likely to engage in specialty care. However, other responses indicate that patients with limited resources need more support than IBHC can provide, while those with sufficient resources may be suitably managed in IBHC.

This split in perspectives emphasizes the competing needs of patients and providers. Patients who providers may feel most confident managing within IBHC (i.e., mild/moderate depression, no comorbidity, high functioning, secure access to resources) may also be those most likely to engage in specialty behavioral health care. However, this may not be the best utilization of specialty care within settings with limited behavioral health resources. Conversely, patients whom providers are least confident they can manage in IBHC (due to acuity, crisis, multiple comorbidities, less access to resources) may be the most in need of IBHC services because of difficulty accessing and engaging in specialty care.

**Fig. 1:**
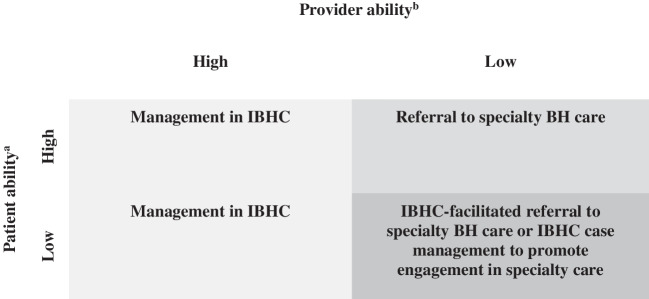
Model heuristic for clinical decision-making: the interaction of patient and provider ability in determining referral to IBHC or specialty behavioral health care. *Notes.* IBHC, integrated behavioral health care; BH, behavioral health. ^a^Patient ability to engage based on resources, history of engagement, preference, condition, or symptom characteristics. ^b^Provider ability to manage patient based on comfort-level managing condition, clinic resources, condition, or symptom characteristics

Without guidance about referrals appropriate and inappropriate for IBHC, providers display heterogeneous perspectives about appropriate referrals, suggesting the need for a heuristic to inform guidance specific to local settings so that referrals to IBHC and specialty behavioral health care may best serve patient and provider needs. Based on the complex interrelationships between provider and patient needs (i.e., ability to manage or engage, respectively), Fig. 1 presents an example model heuristic that could guide standardization and implementation of interventions to support more nuanced decision-making for local, real-world settings moving toward integration of behavioral health care. In the model heuristic, the interaction between patient ability and provider ability guides provider decisions. The model heuristic represents how providers approach referral decision-making in primary care clinics at Boston Medical Center, and its dimensional approach may accommodate the unique needs of various local settings in its conceptualization of patient and provider ability. As such, the model heuristic in Fig. 1 may guide provider training and clinical pathway standardization, in which providers receive specific guidance regarding when to refer patients to IBHC or specialty behavioral health care.^77–80^

For example, patients who demonstrate high ability to engage in care who are evaluated by a provider with limited ability to treat their behavioral health condition (e.g., due to lack of training, limited capacity or resources, lack of training in the appropriate intervention) would likely be referred to specialty care. Patients who demonstrate low ability to engage in care who are evaluated by a provider with high ability to treat them would be indicated for IBHC referral. Patient preference or other clinic specific factors could guide referral decision-making for patients with high ability to engage in care who are evaluated by a provider with high ability to treat them. Finally, cases in which patients with low ability to engage in care are evaluated by a provider with limited ability to provide appropriate behavioral health care may require increased provider training or additional case coordination. Training interventions to increase provider ability to manage conditions associated with high patient need (e.g., serious mental illness, substance use) would improve the ability of IBHC to serve patients who would benefit most.

### Future Directions

To specify how condition characteristics impact a patient's ability to engage and benefit from IBHC, further research on IBHC management of diverse behavioral health conditions with varying levels of acuity and complexity is warranted, focusing on how these variables may moderate IBHC effectiveness. Moreover, future research should leverage mixed method investigation across the condition, patient, and provider characteristics presented here, in both IBHC and specialty care settings, to evaluate IBHC’s effectiveness across the diversity of conditions and symptoms managed in real-world settings.

Future research should also include collaboration with providers in local IBHC settings to further develop tools for referral decision-making standardization that incorporate characteristics most relevant to provider perspectives and patient needs (e.g., Fig. 1). Findings from the present analysis also suggest that increasing provider comfort-level in addressing behavioral health needs could increase the ability of IBHC to manage patients with more challenging and complex behavioral health, if only to help patients get to, and engage in, specialty care. Thus, offering comprehensive and ongoing behavioral health training is essential for expanding the breadth of behavioral health conditions or symptoms managed in IBHC settings.

Moreover, providers and administrator interviews did not speak to the appropriateness of chronic disease and physical health management for referral to IBHC. Some models of IBHC have demonstrated effectiveness in improving indicators of health conditions, like heart disease, diabetes, and chronic pain.^15,17–19,23^ IBHC implementation at Boston Medical Center was limited by a lack of training in health psychology among both primary care and specialty behavioral health providers.^48^ As more clinics implement IBHC models, there are increasing opportunities for managing chronic health conditions. Future research should focus on training needs for provision of behavioral health care for managing physical health concerns.

IBHC settings in which providers have limited training or comfort-level in managing behavioral health are best positioned to address the high patient ability–high provider ability scenario, which limits access to IBHC and could undermine its intended purpose. Thus, clinical and research efforts should focus on implementing and documenting the impact of increased provider training in IBHC across a variety of behavioral health conditions. It is also important to note that current findings highlight the paradox of stepped-care IBHC models: patients who would benefit the most from IBHC’s potential to improve access and engagement are more likely referred to specialty care due to high care needs, while patients with high ability to access specialty behavioral health care may be considered a better fit for IBHC based on level of care needs. The difference in how patient and provider ability are managed in real-world implementation of IBHC versus the stepped-care model championed by some IBHC researchers further supports the need for more nuanced models in real-world and research settings. These models could encourage, for example, the integration of low-intensity interventions into specialty care and high-intensity interventions into IBHC.

### Limitations

Despite the broad implications discussed above, interpretation of these findings is limited by a small sample size, participant self-selection bias, and the uneven distribution of the sample across clinics and participant roles, limiting this study’s ability to compare perspectives between provider types or clinic setting. Nonetheless, sample size was partially determined through theoretical saturation, and sample distribution across roles and clinics is similar to provider and administrator distribution in the local setting.

In addition, the qualitative nature of the present analysis limits the findings to narrative perspectives, which is in line with the researchers’ goal to gain a deep and broad understanding of provider referral. Although the findings include frequency of each subtheme, these should be interpreted with the knowledge that the interviews did not systematically prompt for factors that influenced referral decision-making. Moreover, the present analysis did not aim to assess whether cases were effectively managed in IBHC or in specialty care. Quantitative referral outcome measures could allow future researchers to evaluate real-time referral decision-making and outcomes and inform how standardization may support improved patient outcomes.

The use of “appropriate for IBHC” and “inappropriate for IBHC” represent another limitation. Participants likely used these categories based on the wording of interview questions which used the terms “appropriate” and “inappropriate.” This language is potentially stigmatizing and may not reflect the flexible and interactive nature of referral decisions as described by participants in the present analysis. More inclusive and less rigid language would benefit future research on referral decision-making in IBHC.

Since the collection of these data and in response to researchers’ analyses, several changes have been implemented within the IBHC program of study with the goal of improving the overall success of implementation. Notably, additional stepped-care model-based features have been incorporated to improve referral and engagement in specialty care when needed. In addition, like many institutions, due to COVID-19 there has been massive shifts to telehealth, which has had yet unclear impacts on both IBHC burden and referral practices. Despite limiting generalizability, these changes demonstrate the importance of continual empirical assessment of IBHC implementation to improve adaptation in real-world settings.

## Implications for Behavioral Health

The current analysis aimed to capture provider and administrator perspectives on clinical decision-making in an integrated behavioral health care (IBHC) program within primary care at a large health institution to inform a model guiding patient referral to IBHC versus specialty behavioral health care. Given inconsistent responses regarding conditions and symptoms deemed appropriately, inappropriately, or currently managed in IBHC, providers and administrators would benefit from increased standardization and support in provider clinical decision-making. Diverging from findings demonstrating the broad success of IBHC to address specific conditions, a dimensional approach based on the interaction between patients’ ability to engage in care and providers’ ability to treat them may best fit provider clinical decision-making in low-resource settings newly implementing IBHC that serve diverse patient populations. A heuristic model that considers both provider ability to manage a condition and patient ability to engage in care may be useful in standardizing and supporting provider referral decision-making in IBHC.

## Data Availability

The data used in the current analysis are not available in order to protect the confidentiality of participants.

## Declarations

### Reporting Guidelines

O'Brien BC, Harris IB, Beckman TJ, Reed DA, Cook DA. Standards for reporting qualitative research: A synthesis of recommendations. *Academic Medicine.* 2014;89(9):1245–1251. Available from https://journals.lww.com/academicmedicine/fulltext/2014/09000/Standards_for_Reporting_Qualitative_Research__A.21.aspx. Accessed 7 August 2023.
